# Supplementary figures and images for: Characterization of a composite with enhanced attraction to savannah tsetse flies from constituents or analogues of tsetse refractory waterbuck (Kobus defassa) body odor
Source: PLoS Negl Trop Dis. 2021 Jun 1;15(6):e0009474. doi: 10.1371/journal.pntd.0009474 (PMC8195394; doi:10.1371/journal.pntd.0009474)

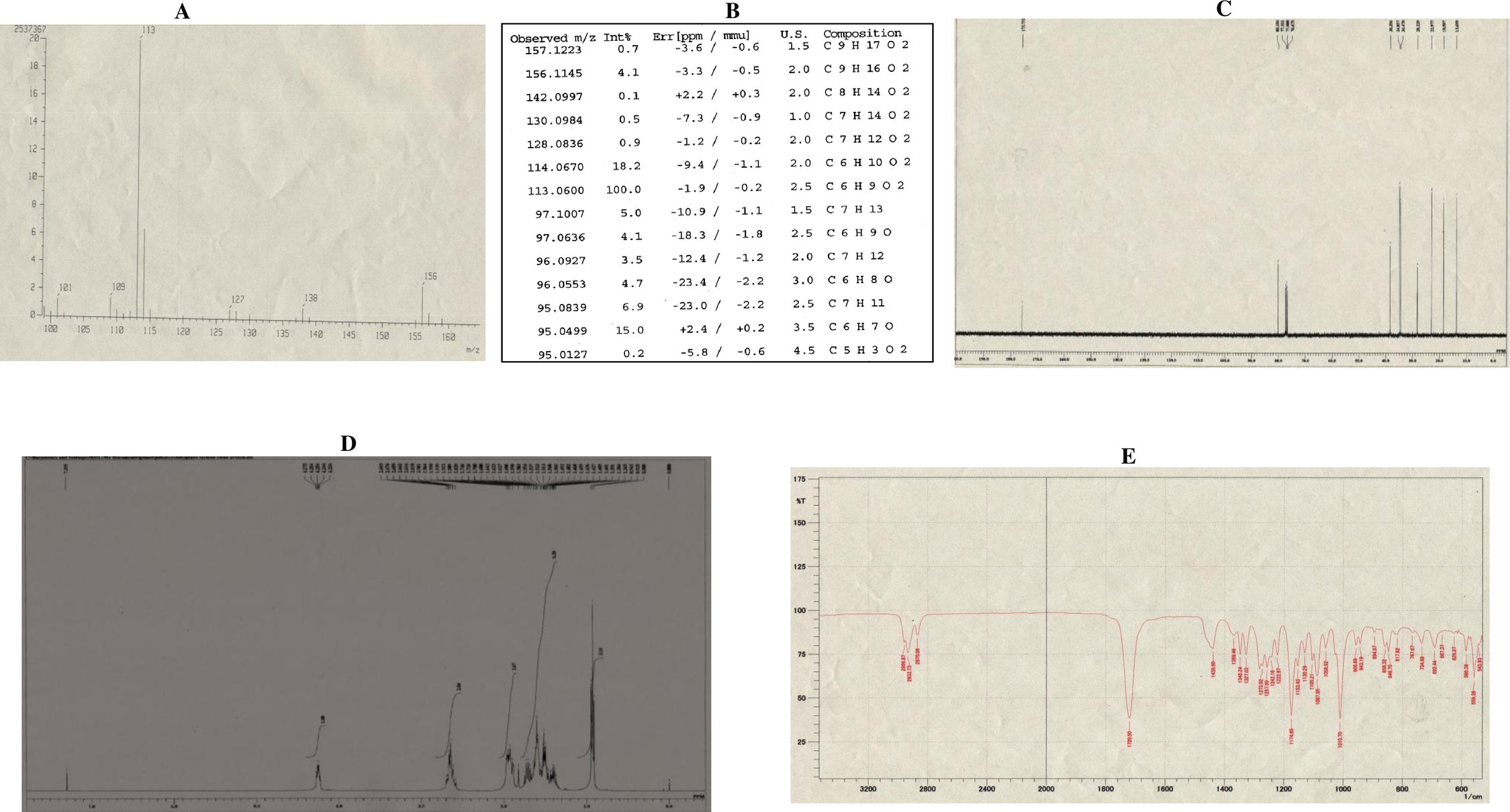

Supplement: S1 Fig — A: High Resolution–Mass Spectrometer spectrum. B: Exact molecular masses leading to identification. C: Carbon-13 Nuclear Magnetic Resonance spectrum. D: Proton (H) Nuclear Magnetic Resonance spectrum. E: Infra-red spectrum. (TIF) [file pntd.0009474.s001.tif]
