# Supplementary material for: Characterization of a composite with enhanced attraction to savannah tsetse flies from constituents or analogues of tsetse refractory waterbuck (Kobus defassa) body odor
Source: PLoS Negl Trop Dis. 2021 Jun 1;15(6):e0009474. doi: 10.1371/journal.pntd.0009474 (PMC8195394; doi:10.1371/journal.pntd.0009474)
Supplement: Attachment — Submitted filename: Plos review.pdf [file pntd.0009474.s002.pdf]

Reviewer 1:

This research evaluates the synergistic effects of known molecules from body odor of waterbuck consisting of delta-octalactone, geranyl acetone, phenols, C5-C10 carboxylic acids and C8-C13 2-alkanones that could be use for tsetse flies control by national programmes, communities, military and wildlife centres. These molecules had already been identified by the same authors Wachira et al 2016 and Bett et al 2015 of this paper earlier. Evaluating them both in the lab and field provides evidence to move forward in terms of using these attractants much as more field studies can be done in different tsetse habitats in the region.

Puzzling question is what would the natural concentration of these molecules in the waterbuck?, what is the proportion of attractiveness to *G. pallidipes*, *G.m.morsitans*? is the attraction the same through the other tsetse species for broader application? Since delta-octalactone, geranyl acetone, phenols were identified as repellent what is their effect is if they are left to work alone without the synergistic effects of C5-C10 carboxylic acids, C8-C13 2-alkanones, delta-octalactone (2-nonanone, octanoic acid and nonanoic acid)?

Abstract

Line 36 *G.m.morsitans* all should be italic

Introduction

Line 79 the equipment can be described at one with mention of "linked" twice in a sentence.
Line 90 remove comma in 4thylphenols
Line 103, in the last part of the introduction, its not clear how they authors hypothesized that increasing attractants consisting of (ε-nonalactone, nonanoic acid and 2-nonanone) would have specific number of folds to attraction to savanna species.

Materials and methods

Three replicates of these experiments where done and all the work was performed according to the recommended. However, the choice of 30 flies of three day teneral tsetse flies is not justified in line 139. Can the authors justify. It's not very clear how the control experiment without any treatment was done
Line 139, there is cage (1), the authors need to clarify on this.
Line 152 to 154, the sentence, "we conducted these experiments and recorded the observation three times for each treatment (Odor and concentration). The sentence could be re-structured to mention the

experiment was replicated three times. Secondly, the word "Odor" change to the lower case.

Line 166 to 167 the formula used, the authors need to quote the reference for this formula.
Line 176, the sentence, "number of generations of the flies that had been colonized which could affected their behavioral phenotype. The authors know that on average tsetse flies can have about 8 generations per year (Krafsur et al 2008). The authors could clarify on how this justify this sentence especially on the behavior and the time this experiment was performed during the year.

Field bioassay validations of wind tunnel for behavioral responses of *G. m. morsitans* and *G. pallidipes*

From line 175 to 223, there is no control or mention of control included in this part of the field experiment. The authors need to mention what controls where used much as they are presented in the result section but could be capture in the materials and methods section.

Results

Line 237 to 241 starting sentence " the structure of ε-nonalactone sounds more of the mention description than results. Possibly this can be taken to the methods and summary results of these techniques summaried.
Line 259 to 262, how was the significant tested??
Line 269 to line 273, in each treatment, there was significant difference which the authors should mention as indicated by the standard error bars in these results description.

Figure, 3B Line 278, I was wondering why the response was increasing gradually in the different treatment? Could the authors clarify why this graph is performing in this way?

Line 503, Table 4: Am wondering why *G.m morsitans* data for the same treatment of *G. pallidipes* is not presented. Could the authors clarify?

Discussion
Line 305, *G. pallidipes* or *G.m morsitans*, authors could use "and"
Line 308 authors could describe more why they are no significant difference in *G.pallidpes* and *G.m. morsitans*.
General comment on the discussion
Much of the results are presented in this paper could be discussed more. I find three paragraph discussion very limiting as compared to the data presented
