# Supplementary material for: Characterization of a composite with enhanced attraction to savannah tsetse flies from constituents or analogues of tsetse refractory waterbuck (Kobus defassa) body odor
Source: PLoS Negl Trop Dis. 2021 Jun 1;15(6):e0009474. doi: 10.1371/journal.pntd.0009474 (PMC8195394; doi:10.1371/journal.pntd.0009474)
Supplement: Attachment — Submitted filename: Review.docx [file pntd.0009474.s003.docx]

Odor blend with enhanced attraction of selected savannah tsetse fly vectors of African trypanosomiasis

This paper shows a promising odor blend that can attract tsetse flies in the field and show some effect in laboratory olfactometer studies. The field data is particularly strong. However, the paper needs to address the comments below before it is ready for publication.

Major issue:

161-163

“we conducted all the exposures in three independent biological replicates in the mornings”

Due to the statistics significance results in Table 1. It is unclear if they are preforming a pseudo replication. Here you use groups of flies as oppose to individuals. By using groups of flies rather than individual, the statistics cannot treat each fly as a replicate, which it appears to. Each groups of flies percent attraction as a whole is a replicate. Avg. should be taken from the 3 replicates, rather than each of the 90 mosquitoes.

Considering this the whole of the results should be statistically reevaluated before publication is warranted.

See:

Ramnirez C.,Contrearas E. Rodriguez L., & Niemeyer H. PSEUDOREPLICATION AND ITS FREQUENCY IN OLFACTOMETRIC LABORATORY STUDIES ( 2000)

Minor Issues:

Line 22 “Savannah tsetse flies void waterbuck (Kobus defassa) due tsetse repellent blend in their body odor “

this sentence doesn’t fully make sense to me, please reword.

Line 43-45“These experiments identified and validated for the first time a novel blend potentially more attractive to G. pallidipes, G. m. morsitans and  probably other savannah tsetse species than their natural host “

The natural host was never tested , the use of Fermented cow urine is not the same to make such claim. “novel blend potentially more attractive to G. pallidipes, G. m. morsitans and  probably other savannah tsetse species than their natural host. “

Line 46-47. “…3.4-folds faster suppression of local populations of these species than conventional tools”.

Population suppression occurs over time is not equivalent to the experiments conducted. What was shown was an increase in trapping ability to the blend over FCU, to suggest otherwise is a reach.

135-137 “We conducted this evaluation using plexi glass cuboidal wind tunnel (195 cm × 136  20 cm × 20 cm) (Figure 1) following protocols we previously described Gikonyo et al 2002 and modified in Wachira et al.,^21^. “

This is vague, which protocol did you use, the original or the modified version ?

Figure 2. Wind tunnel responses of G. pallidipes and G. m. morsitans to attractive blends in varying proportions

Please provide clear description of this result, the metric being measured. It is unclear both in text and figure.

Supplementary Files should be digitalized if possible currently they are difficult to interpret.
