# Supplementary material for: Characterization of a composite with enhanced attraction to savannah tsetse flies from constituents or analogues of tsetse refractory waterbuck (Kobus defassa) body odor
Source: PLoS Negl Trop Dis. 2021 Jun 1;15(6):e0009474. doi: 10.1371/journal.pntd.0009474 (PMC8195394; doi:10.1371/journal.pntd.0009474)
Supplement: Attachment — Submitted filename: Responses to reviewers.docx [file pntd.0009474.s004.docx]

**Responses to reviewers’ comments**

**Reviewer 1:**

**Reviewer:** Puzzling question is what would the natural concentration of these molecules in the waterbuck?, what is the proportion of attractiveness to G. pallidipes, G.m.morsitans? is the attraction the same through the other tsetse species for broader application? Since delta-octalactone, geranyl acetone, phenols were identified as repellent what is their effect is if they are left to work alone without the synergistic effects of C5-C10 carboxylic acids, C8-C13 2-alkanones, delta-octalactone (2-nonanone, octanoic acid and nonanoic acid)?

**Response:** In previous studies, the constituent compounds of waterbuck body odor were identified and consisted of delta-octalactone, geranyl acetone, phenols, C5-C10 carboxylic acids and 2-alkanones. Within each class of compound structure-guided activity studies were carried and most active compound or analogue from each class identified. Subsequently, bioassay guided blending and optimization studies were carried out. In a previous study we reported a four-component repellent blend comprising of δ-nonalactone, heptanoic acid, 4-methylguaiacol and geranyl acetone in 6:4:2:1 (Wachira et al., 2020). We herein report a three-component blend comprising of ε-nonalactone, nonanoic acid and 2-nonanone in 1:3:2.

Abstract

**Reviewer:** Line 36 G.m.morsitans all should be italic

**Response:** Italised *G. m. morsitans*

Introduction

**Reviewer:** Line 79 the equipment can be described at one with mention of

“linked” twice in a sentence.

**Response:** These are two different attenuated equipments; i) Gas chromatography linked with electroantennographic detector, and ii) Gas chromatography linked with mass spectrometer

**Reviewer:** Line 90 remove comma in 4thylphenols

**Response:** Comma removed

**Reviewer:** Line 103, in the last part of the introduction, its not clear how they authors hypothesized that increasing attractants consisting of (ε-nonalactone, nonanoic acid and 2-nonanone) would have specific number of folds to attraction to savanna species.

**Response:** The sentence removed and rephrasing done.

Materials and methods

**Reviewer:** Three replicates of these experiments where done and all the work was performed according to the recommended. However, the choice of 30 flies of three day teneral tsetse flies is not justified in line 139. Can the authors justify. It’s not very clear how the control experiment without any treatment was done

**Response:** For wind-tunnel evaluations of tsetse flies, 10 flies in each replicate are enough to give a conclusive choice (Gikonyo et al., 2003). However, the higher the numbers the stronger the statistical power. The treatments were the test compounds or blends dissolved in dichloromethane solvent. Control arm of the olfactometer consisted dichloromethane solvent. For blank test, dichloromethane solvent was dispensed in the two arms of the olfactometer.

**Reviewer:** Line 139, there is cage (1), the authors need to clarify on this.

**Response:** In figure 1 (olfactometer), the cage is labelled I. This has been rectified to avoid confusion.

**Reviewer:** Line 152 to 154, the sentence, “we conducted these experiments and recorded the observation three times for each treatment (Odor and concentration). The sentence could be re-structured to mention the experiment was replicated three times. Secondly, the word “Odor”

change to the lower case.

**Response:** sentence rephrased.

**Reviewer:** Line 166 to 167 the formula used, the authors need to quote the

reference for this formula.

**Response:** Cited

**Reviewer:** Line 176, the sentence, “number of generations of the flies that had been colonized which could affected their behavioral phenotype. The authors know that on average tsetse flies can have about 8 generations per year (Krafsur et al 2008). The authors could clarify on how this justify this sentence especially on the behavior and the time this experiment was performed during the year. Field bioassay validations of wind tunnel for behavioral responses of G. m. morsitans and G. pallidipes

**Response:** Colony flies are reared under laboratory conditions after pupa is collected from the field. After several multiplications and generations, we are not sure whether the colony flies are really the same as the field flies and whether their responses to odors are the same. For this reason, we carried out the field assays to validate the wind tunnel responses, which showed that colony and field flies responded to the odors in a similar way.

**Reviewer:** From line 175 to 223, there is no control or mention of control included in this part of the field experiment. The authors need to mention what controls where used much as they are presented in the result section but could be capture in the materials and methods section.

**Response:** We have edited this section to clearly indicate the field control as the standard attractant blend control (POCA).

Results

**Reviewer:** Line 237 to 241 starting sentence “ the structure of ε-nonalactone sounds more of the mention description than results. Possibly this can be taken to the methods and summary results of these techniques summaried.

**Response:** Already captured in the method section and since we are presenting spectral data in the supporting files, we have now rephrased.

**Reviewer:** Line 259 to 262, how was the significant tested??

**Response:** The data for *G. pallidipes* and *G. m. morsitans* was separately analyzed, where the mean attractions of the various blends to either species was subjected to ANOVA and means separated by SNK post-hoc analyses. From the rank-transformed data, the order of attraction of the blends and individual compounds to *G. pallidipes* and *G. m. morsitans* was similar.

**Reviewer:** Line 269 to line 273, in each treatment, there was significant difference which the authors should mention as indicated by the standard error bars in these results description.

**Response:** The significant difference is now clearly shown by indicating the *p*-value.

**Reviewer:** Figure, 3B Line 278, I was wondering why the response was increasing gradually in the different treatment? Could the authors clarify why this graph is performing in this way?

**Response:** The bar graphs in figure 3 indicate an increasing pattern. This is a rearrangement of the data from the lowest to highest before plotting the figures. The sorting from lowest to highest mean tsetse catches was separately done for individual compounds and blends.

**Reviewer:** Line 503, Table 4: Am wondering why G.m morsitans data for the same treatment of G. pallidipes is not presented. Could the authors clarify?

**Response:** Field evaluations were carried out in Shimba Hills Game Reserve where *G. pallidipes* population is dominant. *G. m. morsitans* are also there but their numbers were too low to make any statistical comparison. We are planning to carry out field evaluations against *G. m. morsitans* in the fields where they are dominant.

Discussion

**Reviewer:** Line 305, G. pallidipes or G.m morsitans, authors could use “and”

**Response:** This has been rectified

**Reviewer:** Line 308 authors could describe more why they are no significant difference in G.pallidpes and G.m. morsitans.

**Response:** The discussion part was changed wholly as indicated in the final article.

General comment on the discussion

**Reviewer:** Much of the results are presented in this paper could be discussed more. I find three paragraph discussion very limiting as compared to the data presented

**Response:**

The discussion is now fully rephrased to capture structural changes, blending and optimization of the proportions in the constituent blend.

**Reviewer 2**

**Major issue:**

**Reviewer:** 161-163 “we conducted all the exposures in three independent biological replicates in the mornings”Due to the statistics significance results in Table 1. It is unclear if they are preforming a pseudo replication. Here you use groups of flies as oppose to individuals. By using groups of flies rather than individual, the statistics cannot treat each fly as a replicate, which it appears to. Each groups of flies percent attraction as a whole is a replicate. Avg. should be taken from the 3 replicates, rather than each of the 90 mosquitoes.

Considering this the whole of the results should be statistically reevaluated before publication is warranted.

See:

Ramnirez C.,Contrearas E. Rodriguez L., & Niemeyer H. PSEUDOREPLICATION AND ITS FREQUENCY IN OLFACTOMETRIC LABORATORY STUDIES ( 2000)

**Response:** In each test, thirty flies were introduced into the olfactometer as a group but the behavior of each fly was observed and recorded independently for 3-min. The average distance travelled by flies on exposure to treatments was obtained from individual fly final resting position after three minutes. We were also able to calculate the repellency of tsetse on exposure to treatments from their proportions in the treated and control arms using the formula (Ⓣ-Ⓒ)/(Ⓣ+Ⓒ))*100; where Ⓣ and Ⓒ represents the number of flies in the treated and control arms respectively (Gikonyo et al., 2003; Wachira et al., 2020). Three sets of replicates each evaluating thirty flies were carried out. The data presented are means of 30 x 3 flies.

**Minor Issues:**

**Reviewer:** Line 22 “Savannah tsetse flies void waterbuck (Kobus defassa) due tsetse repellent blend in their body odor “this sentence doesn’t fully make sense to me, please reword.

**Response:** The sentence “Savannah tsetse flies void waterbuck” rephrased to read “Savannah tsetse flies avoid flying toward thick-skinned tsetse-refractory waterbuck”

**Reviewer:** Line 43-45“These experiments identified and validated for the first time a novel blend potentially more attractive to G. pallidipes, G. m. morsitans and  probably other savannah tsetse species than their natural host “ The natural host was never tested, the use of Fermented cow urine is not the same to make such claim. “novel blend potentially more attractive to G. pallidipes, G. m. morsitans and  probably other savannah tsetse species than their natural host. “

**Response:** Fermented cow urine has phenolic blends and together with POCA (P = 3-n-propylphenol; O = 1-octen-3-ol; C = 4-methylphenol; A = acetone) represent synthetic cattle odour. We herein reported a more effective blend than this control. In the statement “….a novel blend potentially more attractive to *G. pallidipes*….” We hypothesized that the blend would be more effective than the real host. We are in the process of planning on carrying out this part of the study.

**Reviewer:** Line 46-47. “…3.4-folds faster suppression of local populations of these species than conventional tools”. Population suppression occurs over time is not equivalent to the experiments conducted. What was shown was an increase in trapping ability to the blend over FCU, to suggest otherwise is a reach.

**Response:** Since our reported blend is 3.4 times as more effective than the control, and that it attracts flies 3.4 times more, we concluded of that the suppressions would be in a similar manner. This statement has been removed and rephrased to avoid confusion.

**Reviewer:** 135-137 “We conducted this evaluation using plexi glass cuboidal wind tunnel (195 cm × 136  20 cm × 20 cm) (Figure 1) following protocols we previously described Gikonyo et al 2002 and modified in Wachira et al.,^21^. “This is vague, which protocol did you use, the original or the modified version ?

**Response:** The modifications was on the olfactometer where the design was cylindrical which was changed to cuboidal as previously described by Wachira et al ^21^. The statement has been rephrased to read “….responses of the flies to individual chemicals and each of the four blends were monitored in a plexi glass cuboidal wind tunnel (195 cm × 20 cm × 20 cm) (Figure 1) following protocols previously described (Gikonyo et al.^9^; Wachira et al.^21^)”

**Reviewer:** Figure 2. Wind tunnel responses of G. pallidipes and G. m. morsitans to attractive blends in varying proportions

Please provide clear description of this result, the metric being measured. It is unclear both in text and figure.

**Response:** The y-axis of figure 2 rephrased to read “Mean % Attraction of tsetse flies”
