# Supplementary material for: Characterization of a composite with enhanced attraction to savannah tsetse flies from constituents or analogues of tsetse refractory waterbuck (Kobus defassa) body odor
Source: PLoS Negl Trop Dis. 2021 Jun 1;15(6):e0009474. doi: 10.1371/journal.pntd.0009474 (PMC8195394; doi:10.1371/journal.pntd.0009474)
Supplement: Attachment — Submitted filename: Responses to Reviewers-Final....docx [file pntd.0009474.s005.docx]

**Responses to reviewers**

**Reviewer:** The work explored development and evaluation of the alternative attractants for savannah tsetse flies which will go a long way in enhancing traditional trapping technologies for these groups of the tsetse flies. The data presented if good for G. pallidipes. I recommend field bioassay are done on the G. m. morsitans to support the findings from this study.

**Response:** We concur with the reviewer, and in our initial design, we did monitor levels of G. m. morsitans catches in our traps. However, the populations of this species are insignificant in numbers of within the East African region as we have indicated in line 173 “We limited our field validation *G. pallidipes,* excluding *G. m. morsitans* that was not present in significant numbers, in tsetse habitats in our region (East Africa). The field *G. m. morsitans* was therefore not available to us for validation at that point.…” Based on our wind tunnel assays and previous responses of the two species to POCA blend and constituents, *G. pallidipes* and *G. m. morsitans* show similar responses to host odors and we expect comparable patterns of catches in the field. However, this needs to be confirmed in the field and we are reaching out to our collaborators in Zimbabwe in southern Africa and elsewhere where G. m. morsitans are naturally abundant for such validation. We have indicated this aspect as a follow study in line 348 “…*Finally, field comparison of the G. pallidipes and G. m. morsitans, where both species co-occur in significant numbers, needs to be undertaken to confirm the laboratory responses of the species, or reveal any subtle differences, especially where both flies are sympatric, which was not possible in the current study*….”

**Abstract**
**Reviewer:** Line 24 to 25, it should be stated that “The carboxylic acids and alkanones” are also derived from waterbuck

**Response:** We thank the reviewer for observation of this omission for which we apologize. We concur and have indeed rephrased the sentence to read “…..*some waterbuck constituents (particularly, nonanoic acid and 2-nonanone) showed*….” (Line 24)

**Reviewer:** Line 25, A structural analogue of the δ-octalactone (ε-nonalactone) is also attractive to the “savanna” species of tsetse flies.

**Response:** We again concur with the reviewer and have rephrased line 26 to confirm this statement; it now “…*In another study, increasing the ring size* *of δ-octalactone from six to seven membered ring changed the activity of the resulting molecule (ε-nonalactone) on the savannah tsetse flies from repellency to attraction*….”

**Reviewer:** Line 32, It’s stated G. pallidipes are naturally abundant in Shimba Hills game reserve.  It is clear the result presented support attraction of G. pallidipes to the new blends. The question how did the authors model this to reflect G. m. morsitans since these two tsetse types are different?

**Response:** We thank the reviewer again for this observation. As we have stated above, it was technically impossible for us to validate G. m. morsitans in the field in shimba hills ow ant where in Kenya of East Africa in general, since this species is not available in this region. We only have access to a laboratory colony. However, field studies by Glyn Vale’s group in Zimbabwe, where both species (G. pallidipes and G. m. morsitans) are sympatric have shown that both species exhibit (to a great extent) similar responses to odor cues, an observation that appears to be supported by response of both species to our odors in the laboratory. We cannot however rule our subtle difference in the responses to odors, hence our indication above that we are engaging our collaborators in Zimbabwe for this evaluation in future. We have indicated this aspect as a follow study in line 348 which reads “…*Finally, field comparison of the G. pallidipes and G. m. morsitans might confirm the laboratory responses or reveal subtle differences, especially where both flies are sympatric, which was not possible in the current study*….”

**Introduction**

**Reviewer:** Line 67 to 68, the authors should quote the references source of bait technologies.

**Response:** We thank the reviewer for pointing out this omission to us for which we apologise. We have now referenced the source of bait technology in line 68 which reads “…. *Artificial visual-olfactory bait technologies have shown significant promise in tsetse fly control operations****^1-3^***…...”

**Reviewer:** Line 97-to 101, needs more specific information to waterbuck odor as shown in POCA justification.

**Response:** We deleted “*comprising of δ-octalactone, phenols (guaiacol and carvacrol), and homologues of carboxylic acids (C5-C10) and 2-alkanones (C8-C13)*” for clarity, flow and also this had been previously mentioned. Line 100 now reads “…. *The present study was built on results from two sets of studies with constituents of tsetse-refractory waterbuck. First, wind tunnel*……….”

In addition, more specific information on composition of waterbuck body odor is stated in line 21 – 23 “…. *comprised of* *δ-octalactone, geranyl acetone, phenols (guaiacol and carvacrol), and homologues of carboxylic acids (C_5_-C_10_) and 2-alkanones (C_8_-C_13_)*….”

**Materials and methods**

**Reviewer:** Line 110, racemic mixture of ε-nonalactone, it might be better to state the proportion of the racemic mixture without referring it to Wachira et al., since this is serious experiment
**Response:** We have clarified in line 111 that “…. *The ε-Nonalactone synthesized consisted of a racemic mixture of equal quantities (50: 50) of (+) ε-nonalactone and (-) ε-nonalactone enantiomers Wachira et al.^21^*.”. We thank the reviewer for pointing this out for us.

**Laboratory bioassays**

**Reviewer:** Tsetse flies used to evaluate the responses of G. m. morsitans where from the insectary

**Response:** We indeed only used insectary G. m. morsitans in our study and we apologise if this did not come our clearly… we have now better clarified in line 131”…*We conducted laboratory studies with both sexes of adult G. m. morsitans or G. pallidipes obtained from colonies maintained at the insectary at Biotechnology Research Institut*e…” and line 261 : “*We limited our field validation to G. pallidipes, excluding G. m. morsitans that was not present in tsetse fly habitats in our region (East Africa). The field G. m. morsitans was therefore not available to us for validation at that point*….” We hope this better clarifies that we only used the G. m. morsitans in the laboratory but not field studies.

**Field bioassay**

**Reviewer:** Line 205, Our sites had similar G. pallidipes densities, would be possibly to state these densities??

**Response:** We thank the reviewer again for poiting out our omission for which we apologise. We have now clarified the average density (fly trap density) of G. pallidipes in line 338 as “…*The sites had similar level of G. pallidipes densities of 200 - 240 flies/trap/da*y...”

**Reviewer:** Line 210, they author could also state the release rates

**Response:** Many thanks again for the suggestion. We have now captured the release rates in line 339 “…*We separately baited each trap on site with fermented cow urine (FCU) (≈ 1000mg/h), acetone (≈500mg/h) and a single odor or blend (19.33 - 22.00mg/h), or no odors (control)*...”

**Results
Lab bioassays**
**Reviewer:** Line 236 to 241, racemic mixture of ε-nonalactone ratios are not shown

**Response:** We have clarified in line 131 that “…. *The ε-nonalactone synthesized consisted of a racemic mixture of equal quantities (50: 50) of (+) ε-nonalactone and (-) ε-nonalactone enantiomers…*”. We thank the reviewer for pointing this out for us.

**Reviewer:** Line 250 Results for flight distance measurement, it is not very clear in the materials and methods how the flight distance was recorded.

**Response**: We measured the flight distance measurements on graduations (in cm) from mid-section of wind tunnel to either ends of the wind tunnel. We have made this clarification in line 194 “… *final landing and resting positions distance (in cm) from the midsection release point in either direction (control or treated arms) in the wind tunnel). Each arm of the wind tunnel was graduated in cm from the midsection to facilitate measurement of flight distances*…”

**Reviewer:** Line 269 to 273, it is not clear what residual solvent was used to get the results shown in Figure 2?

**Response:** We again apologize for this lack of clarity. We have now clarified that the residual solvent used was dichloromethane which we have now stated as in line 190 – 192 “….. *at concentrations of 0.05, 0.25 and 0.5 mg of each compound or blend in 1 ml dichloromethane solvent. We separately released the prepared compounds/blends from one side of the olfactometer and dichloromethane solvent from*….”

**Field bioassays**

**Reviewer:** Line 278, G. pallidipes, G. austeni and G. brevipalpis were attracted. When one checks on the results, there are no results for G. austeni and G. brevipalpis, What do they authors say about this???
**Response:** We again apologize for this omission, G. pallidipes, G. austeni and G. brevipalpis exist in sympatry at Shimba Hills game reserve with G. pallidipes constituting most (> 95%) of the fly populations in our study sites. Consequently, the responses (numbers) of G. austeni and G. brevipalpis were about 12 and 4 flies per trap per day respectively, which were insufficient densities for us draw relevant statistical inference regarding their responses to our treatment. We thus excluded them from further analyses. We have now made this clarification in line 443 - 456 of the manuscript “…*The G. pallidipes, G. austeni and G. brevipalpis are sympatric at Shimba Hills game reserve, with G. pallidipes most predominant, constituting 95.42 % of the overall numbers of flies that responded to treatments. The G. austeni and G. brevipalpis constituted 2.83 and 0.49 % respectively. Consequently, the responses of G. austeni and G. brevipalpis were about 12 and 4 flies per trap per day, which we considered insufficient for us to draw relevant statistical inference regarding responses of the respective species to our treatment. We thus excluded these two species from further analyses.*..” We are very sorry for the oversight of this important information.

**Discussion**

**Reviewer:** Line 308 to 309, the authors could expand on the similar receptors and possible genes, if there is some literature in this line. Similar to line 320 to 321

**Response:** We thank the reviewer for the suggestions. However, research on olfactory receptors in tsetse flies in still nascent. We thus elected to leave this out of our discussion since most of the available information is putative/speculative and none of them touch on our specific compounds/blends.

**Reviewer:** Line 311, subtle structural changes, possibly the authors could look at one thing at a time of increase assessment of the aliphatic and also separately decrease with supporting data. Do authors have these data in the results?

**Response:** Different classes of compounds from the waterbuck odor had different structure guided studies. For instance, structure-activity studies of lactones concentrated on ring sizes, length of the side chain and electron densities while for the ketones and alkanones is only on chain length. In our discussion, we generalized all the changes and we could not separate them as shown in line 529 which reads “…..*confirm that very subtle structural changes in some of the constituents of waterbuck body odour (lactone, 2-alkanone and carboxylic acid) are*…..”

**Reviewer:** Line 326, The authors have to remember that there was no data for G. austeni and G. brevipalpis populations to support this statement.

**Response:** We thank reviewer for this suggestion and have consequently expunged statements on G. austeni and G. brevipalpis populations in the current version. We again apologize for this inclusion in the previous version.

**Reviewer #2:**

The authors’ presentation of the discovery of an attractive components of an otherwise repellent odor blend from the Waterbuck is compelling and worth publishing. The combination of laboratory and field work strengthens the conclusions of the paper and could be applied to Tsetse fly control. I do have some suggestions for revision to clarify the manuscript and make it more accessible to a wider readership. The authors have adequately addressed the comments by previous reviewers.

**Key points to revise:**

**Reviewer:** The abstract could be more concise and more clearly state the importance of the study. The author summary is clearer than the abstract and highlights the finding better for the reader. The abstract should be revised, and the author summary should inspire the revised text.

**Response:** We than the reviewer for this important suggestion. We have consequently revised the abstract to capture the importance of the study as suggested.

**Reviewer:** Line 106: The final sentence is unnecessary, please remove it “Herein we report our findings”

**Response:** We have removed the offending sentence as suggested. We do apologize for the inclusion.

**Reviewer:** Line 343-344: The sentence “The results of these studies will be reported elsewhere” should be removed. It is unnecessary and does not add anything to the article. Better to add a concluding sentence discussing the relevance of the work towards the development of better attractants for Tsetse flies.

**Response**: We have removed the sentence “*The results of these studies will be reported elsewhere*” and concur that it is unnecessary. We have additionally added a concluding statement in line 555 “…. *The results of this study lay down useful groundwork in the large-scale development of more effective tsetse baits to be used in ‘pull’ and ‘push-pull’ control tactics*” as suggested.

**Figures:**
**Reviewer:** Figure 1 & 2 can be combined so it is clear that figure 2 data arises from the assay depicted in figure 1.

**Response:** We have combined figures 1 and 2 and labelled them as panels 1A and 1B as suggested.

**Reviewer:** Figure 2: Y axis should be set from 0 to 100%. The baseline attraction in the assay to the residual solvent is obscured. Using a dot scatter plot instead of a box plots would more easily allow the reader to assess the N used in each treatment. Color could be used to indicate species. The results are significant and important, but full transparency is necessary for the reader to properly interpret the data.
**Response:** The lowest mean percentage attraction of tsetse to the odors was >50% and the scale was cut to start at 50% for a clearer view of the differences. We have introduced diagonal lines on the y-axis to indicate a scale break.

**Reviewer:** Figure 3: The resolution of the figure 3 is poor, please use a higher resolution file. A diagram of the field experimental set up would be useful as an additional panel A. This would allow the reader to clearly differentiate between laboratory and field data. Even though it is described clearly in the text, a graphical representation will help the reader understand the importance of the results here. This is a critical figure that presents crucial data that supports the authors hypothesis, and it needs to be highlighted in the manuscript.

**Response:** We have replaced with a clearer figure. We have added a diagram of the field experimental set up and labelled it as panel 2A as suggested by the reviewer.
